# Academic Stress Interventions in High Schools: A Systematic Literature Review

**DOI:** 10.1007/s10578-024-01667-5

**Published:** 2024-03-04

**Authors:** Tess Jagiello, Jessica Belcher, Aswathi Neelakandan, Kaylee Boyd, Viviana M. Wuthrich

**Affiliations:** Lifespan Health & Wellbeing Research Centre, Macquarie University, Sydney, Australia

**Keywords:** Academic stress, Test anxiety, High school, School-based programs, Psychological interventions

## Abstract

The experience of academic stress is common during high school and can have significant negative consequences for students’ educational achievement and wellbeing. High school students frequently report heightened levels of school-related distress, particularly as they approach high-stakes assessments. Programs designed to reduce or prevent academic stress are needed, and their delivery in school settings is ideal to improve treatment access. The current review aimed to examine the effectiveness of high school-based programs in reducing or preventing academic stress. A systematic search returned 31 eligible studies across 13 countries. Programs were categorised according to intervention type, format, and facilitator. Results showed that the methodological quality of most studies was poor, and many used an inactive control group. As predicted by theories of academic stress, the strongest evidence was for programs grounded in cognitive-behavioural therapy (CBT). There was evidence that both universal and targeted approaches can be beneficial. The unique implementation issues for these two formats are discussed. Most programs were delivered by psychologists and were generally effective, but almost all of these were CBT programs. A smaller proportion of programs delivered by teachers were effective. Therefore, future studies should evaluate the implementation success of programs to improve the rate of effective delivery by school staff. Overall, the field will benefit from more randomised controlled trials with comparisons to active control groups, larger sample sizes and longer-term follow-ups.

## Introduction

Academic stress is defined as the transient experience of pressure, anxiety, or distress related to achieving academic goals [1]. Theoretically, students experience academic stress when they are concerned about their capacity to cope with academic challenges [2]. Test anxiety, which originally was narrowly defined as the fear of taking tests or exams [3], has been shown to strongly overlap with academic stress [4] and anxiety disorders [5], and as such, most of the research on academic stress has come from literature examining test anxiety. This research has consistently shown that test anxiety is comprised of two components: academic-related worry (intrusive and repetitive thoughts about failing) and emotionality (emotional distress and physiological tension) (see [6]. Models of test anxiety (e.g. [7, 8] predict that cognitive factors (such as negative self-beliefs, low self-efficacy, and appraisal of situations as threatening) and unhelpful study behaviours (such as avoidance and sabotage) are important factors in maintaining academic anxiety. Consistent with this, structural equation modelling has shown that high school students with more realistic cognitions, higher academic self-efficacy, and better coping strategies in response to stress experienced less test anxiety and performed better in their examinations [9]. A recent review of 60 studies found that similar factors were related to increased academic stress among high school students, including higher trait anxiety, worry about failure, perfectionism, avoidant coping, and lower academic confidence and resilience [4].

Addressing academic stress in school students is important given its potentially serious impact on educational attainment and wellbeing. High levels of academic stress are associated with poorer examination performance, mental wellbeing, affect, sleep, confidence, motivation, and even physical health [1, 10–13]. In samples of Australian high school students, severity and prevalence of academic stress is a cause for concern. Academic anxiety has been shown to be significantly higher for students in later high school grades relative to lower grades [14, 15], particularly when students are faced with “high-stakes” examinations at the end of high school education that are associated with entrance ranks into university courses [16–19]. National and international research has revealed that coping with school-related stress is a primary concern for high school students [20], with approximately 20% reporting very high levels of stress in the final years of high school [4, 21], which increases throughout the final year of school [21]. Taken together, this data highlights the need for interventions to target academic stress specifically in high school students who are faced with high-stakes assessments and who experience increasing levels of stress.

Academic stress interventions can be made more accessible by delivering programs in the school setting [22, 23]. School-based programs are endorsed by the school, delivered on school grounds, during class time, or as an after-school activity, and can be run in group or individual format. Universal programs are delivered to an entire class or grade (usually as part of the school curriculum) regardless of whether students are currently experiencing distress. Therefore, universal programs are sometimes referred to as a “preventative” approach. Targeted programs can be selective or indicated, which are provided to students at increased risk of developing an anxiety disorder (i.e., with particular risk factors) or to students with symptoms of distress (i.e., scoring above a cut-off on a self-report measure, or identified by school staff as distressed).

To date, previous literature reviews have not examined the effectiveness of school-based interventions to reduce academic stress in high school students. Some reviews have been conducted to evaluate mental health promotion or prevention programs (for a range of mental disorders) in schools, however they did not focus exclusively on programs designed to target academic stress [22, 24–30]. A small number of reviews have investigated the effectiveness of test anxiety/stress-management interventions for school-aged children, but none specifically in high school students [31–33]. Common findings across these reviews were that targeted and universal programs have both led to reduced distress, programs were most often delivered by trained professionals (but some had been successfully delivered by teachers), and programs teaching cognitive-behavioural strategies were the most studied and had the strongest evidence for reducing anxiety.

A specialised review of the literature is needed on whether programs specifically for academic-related stress can be implemented effectively in schools for high school students, as this is the peak period for academic stress. To the best of our knowledge, this is the first systematic review to focus exclusively on school-based programs for academic stress (including, but not limited to, test anxiety) in high school students. The aim of the current review was to better understand which types of interventions are effective and the characteristics that may alter effectiveness. From previous literature and models of academic stress, it was hypothesised that interventions which targeted theorised and known factors contributing to heightened stress (such as negative thoughts and unhelpful coping styles) would be most likely to be effective. It is anticipated that the results from this review will assist schools to select evidence-based programs that help students better manage the demands and stresses of high school.

## Method

### Search Strategy

A systematic literature search was conducted using the databases, PsycINFO (American Psychological Association) 1806 to March 2023 and Education Resources Information Centre (ERIC; Institute of Education Sciences) 1966 to March 2023. Keywords were developed to capture school-based stress reduction or prevention programs for high school students: [program OR intervention OR training OR promotion] AND [stress or anxiety or academic stress or test anxiety] AND [school OR high school OR secondary school OR senior school OR school based OR classroom] AND [student* OR adolescent* OR child*] AND [target* OR universal OR at risk OR prevent* OR reduction OR reduce]. Results were limited to studies published in English peer-reviewed journals. This search returned a total of 2,871 articles.

### Inclusion and Exclusion Criteria

Studies had to meet the following criteria to be included: (a) participants were high school students; (b) the intervention was school-based; (c) the intervention primarily aimed to reduce or prevent school-related stress or anxiety (i.e., the authors specifically stated that the intervention targeted academic or school-related stress, the authors noted details of the intervention that included reference to academic or school-related stress or the authors included a measure of academic or school-related stress pre- and post-intervention); (d) a primary outcome measure included students’ level of stress or anxiety, measured at both pre- and post-intervention; (e) the intervention group was compared statistically to a control group in a randomised controlled trial. All types of interventions were eligible (e.g., physical, psychological, educational) and the intervention could either be targeted or universal. Given that Australian high schools include grades 7 to 12, studies that reported on “middle school” students in grades 7 or above were included. Similarly, studies that included both primary and high school students were included if they reported subgroup analyses for high school students. Studies were excluded if they did not meet the inclusion criteria, or if: (a) subgroup analyses were not reported for high school students; (b) the intervention primarily aimed to reduce or prevent non-academic stress or an anxiety disorder (e.g., posttraumatic stress disorder, social anxiety disorder), mood disorder, or a problem behaviour (e.g., drug use, truancy); (c) the study was a review or research protocol.

### Study Selection and Data Extraction

**Fig. 1:**
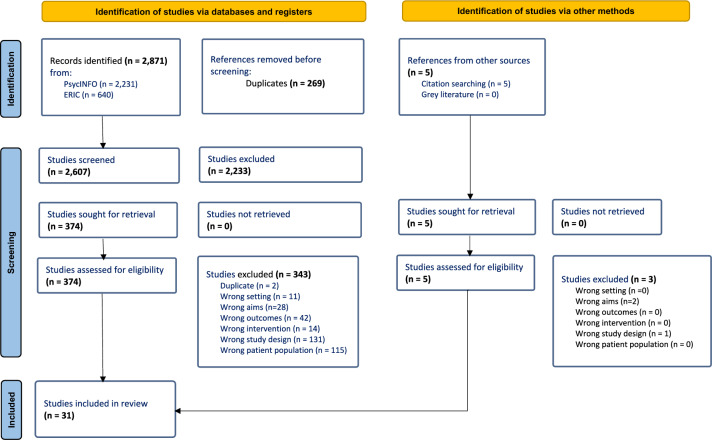
PRISMA flow diagram

Using Covidence Systematic Review Management Software, articles were screened first by their title and abstract, and then by full text with regards to the above criteria by two authors (TJ, JB). From the database search, 31 articles met the eligibility criteria and were included in this review. An additional 5 articles sourced from reference lists were also found to be relevant, however only 2 met inclusion criteria and were included [34, 35]. Details regarding the study selection process are reported in Fig. 1, based on the PRISMA guidelines [36]. The following data was extracted for the 31 included articles, which can be found in Table 1 (targeted interventions) and Table 2 (universal interventions): (a) sample characteristics including size, age, grade, school and country; (b) intervention and comparison group; (c) program format (i.e., targeted or universal) and treatment type; (d) program facilitator and number of sessions; (e) outcome measures of distress; (f) summary of results related to stress or anxiety including effect size. Studies in which students self-selected to engage in a program (but did not require a set level of symptoms) were considered targeted interventions, as the students likely believed it would be helpful to reduce symptoms.

**Table 1:** Targeted interventions included in the review

Author	Setting	Overall sample (N)	Age range/ Grade	Intervention and Comparison groups (n)	Format & treatment type category	Facilitator(s) & number of treatment sessions	Stress/Anxiety Outcome Measure(s)	Results	Findings Summary
1. de Hullu et al. [37]	24 Dutch schools	240	12 – 16 years Grade not reported	1. Internet-based CBM training (n = 86) 2. School-based CBT (n = 84) 3. No intervention control (n = 70)	Targeted – high social anxiety at school Cognitive bias modification -computer based tasks to modify interpretation and attention biases, strengthen association between social evaluative situations and positive outcomes and increase implicit self-esteem	Psychologist 20 sessions (2 sessions per week)	1. Revised Child Anxiety and Depression Scale – Social phobia subscale [38] 2. Spielberger Test Anxiety Inventory (TAI)	Significant decrease of TAI scores, p < 0.0001, d = 0.82 (CBT) and d = 0.65 (CBM) and RCADS scores, p < 0.0001, d = .86 (CBT) & d = 0.79 (CBM). Intervention group interaction not significant, p = 0.32	Test anxiety and social anxiety significantly reduced from pre-treatment to 2-year follow-up across all conditions, there was no difference between CBM, CBT or no-intervention control
2. Gaesser & Karan [39]	10 American schools	63	10—18 years. Grades 6–12	1. CBT group (n = 21) 2. Emotional Freedom Technique (EFT) group (n = 21) 3. Waitlist control (n = 21)	Targeted – high ability students with school based anxiety CBT -cognitive reframing -building awareness -systematic desensitisation	Professional instructors 3 sessions	1. Revised Children’s Manifest Anxiety Scale-2 (RCMAS-2)	Students in EFT group had significantly lower post anxiety (p = 0.005, d = 0.34	Anxiety significantly decreased pre- to post-intervention for both the EFT and CBT groups. However, only EFT change in anxiety symptoms pre- to post-intervention was significantly higher compared to control
3. Hains & Ellman [40]	1 American school	21	Age not reported Grades 9, 10, 11, 12	1. Stress-inoculation training (n = 11) 2. Waitlist Control (n = 10)	Targeted – self selected CBT -cognitive restructuring -problem-solving -relaxation	Psychologists 13 sessions	1. State-Trait Anxiety Inventory (STAI; [83]) 2. Adolescent Perceived Events Scale (APES)	Significant trait anxiety reductions at follow-up in high emotional arousal group. T(5) = 3.13, p = 0.013. No effect size given	No significant group differences in anxiety or stress at post-intervention. At follow-up, trait anxiety significantly reduced for students with “high emotional arousal” (scored above a cut-off at baseline) who received the intervention
4. Johnson et al. [41]	1 American school	30	Age not reported Grade 8	1. Systematic desensitisation (n = 8) 2. Speech practice (n = 8) 3. No treatment control (n = 8)	Targeted – high speech anxiety Systematic desensitisation -relaxation and visualisation -speech fear hierarchy -script for desensitisation related to fear hierarchy	Psychologist 9 sessions	1. Speech Anxiety Survey (researcher adapted)	Significant anxiety drops in intervention groups (D: p < 0.01; P: p < 0.01). No effect size given	Significantly reduced anxiety for students in both intervention groups, relative to control. No significant difference between interventions
5. Kamour & Altakhayneh [42]	Jordanian private schools	207	Age not reported Grade 7 & 8	1. SEL program (n = 100) 2. Control group (n = 107)	Targeted – unknown “deliberately chosen” Social Emotional Learning -self awareness -self management -social awareness -relationship skills -responsible decision making	Not reported 16 sessions	1. Math Anxiety Test (researcher designed)	Reduction of math anxiety. (F = 4413, p = 0.000)	Significant between group difference indicating the SEL group had a greater reduction in math anxiety compared to the control group pre- to post-intervention
9. Keogh et al. [34]	1 English school	209	15—16 years Grades not reported	1. Cognitive-behavioural stress management intervention (n = 80) 2. No intervention control (n = 80)	Universal CBT -psychoeducation -challenging unhelpful cognitions -progressive muscle relaxation -modify meta-cognitions about worry -adaptive problem solving -guided imagery	Psychologist 10 sessions	1. Revised Test Anxiety Scale (RTA; [43] 2. General Health Questionnaire (GHQ) – Mental ill-Health 3. GCSE points	No significant main, or interactive, effects	No significant changes in test anxiety Mental ill-health improved from pre- to post-intervention for the CBT group but not the control group The CBT group obtained higher GCSE scores compared to the control group, with the CBT group obtaining one letter grade (i.e., B grade) higher than the control group (i.e., C grade)
10. Khalsa et al. [44]	1 American school	121	15—19 years. Grades 11 & 12	1. Yoga (n = 74) 2. Usual Physical Education class (n = 47)	Universal Mindfulness/ Meditation -training in cognitive skills of mindfulness and self-awareness -yoga based psychological attitude -yoga poses -breathing exercises -visualisation -relaxation -stress management	Professional instructor 11 weeks, 2–3 sessions per week (i.e., 23–32 sessions dependent on class)	1. Perceived Stress Scale (PSS) 2. Profile of Mood States-Short Form (POMS-SF) 3. Behaviour Assessment Survey for Children-Version 2) – includes test anxiety scale	No significant group differences (p = 0.15)	No significant group differences for anxiety or stress pre- to post- intervention
11. Kiselica et al. [45]	1 American school	48	Age not reported Grade 9	1. Stress inoculation training (n = 24) 2. Active control (n = 24)	Universal CBT -psychoeducation -progressive muscle relaxation -cognitive restructuring -assertiveness training	Psychologists 8 sessions	1. State-Trait Anxiety Inventory – Trait (STAI A-TRAIT; [85]) 2. Symptoms of Stress Inventory (SOSI; [82]) 3. GPA	Significant dif. between treatment and control participants (F(1,41) = 14.04, p < 0.001)	Significantly lower anxiety and stress for students in the intervention condition compared to the control condition at post-treatment and follow-up. No significant differences between groups for GPA
12. Lang et al. [46]	1 Swiss vocational school	131	Mean age 16.22 years (range, grade not reported)	1. PE based coping training -EPHECT (n = 67) 2. No intervention control (n = 64)	Universal CBT -psychoeducation -emotion and problem focussed coping skills	Teachers 8 sessions	1. Adolescents Stress Questionnaire (ASQ; [47]	Time by group interactive effect at follow- up (F(1,111) = 3.82, p < 0.05)	Significantly greater reduction in stress for the intervention group, relative to controls, at 6-month follow-up
13. Lang et al. [48] *Same sample as 2016	1 Swiss vocational school	131	Mean age 16.22 years Age range and grade not reported)	1. PE based coping training -EPHECT (n = 67) 2. No intervention control (n = 64)	Universal CBT -psychoeducation -emotion and problem focussed coping skills	Teachers 8 sessions	1. Adolescents Stress Questionnaire (ASQ; [47] 2. Coping Questionnaire for Children and Adolescents (SVF-KJ)	No significant changes pre-post treatment	Intervention group reported greater increase in emotion focused coping compared to control group pre-post intervention No significant changes in perceived stress from pre- to post-treatment
6. Laxer & Walker [49]	3 Canadian schools	119	Age not reported. Grade not reported	1. Systematic desensitisation 2. Relaxation alone 3. Simulation alone 4. Relaxation plus simulation 5. Attention control 6. No treatment control *N in each group not reported	Targeted – high test anxiety Systematic desensitisation -deep muscular relaxation -presentation of graded imaginal stimuli associated with examinations	Professional instructors 20 sessions	1. Alpert-Haber Achievement Anxiety Scale (AAS) 2. Taylor Manifest Anxiety Scale (TAS)	Significant difference only for test anxiety measure. (F = 4.14, p < 0.001)	Test anxiety (TAS) was significantly lower for students in the systematic desensitisation condition and relaxation alone condition, relative to no treatment control
7. Lowe & Wuthrich [83]	4 Australian schools	56	17 – 18 years. Grade 12	1. Study Without Stress (SWoS) program (n = 28) 2. Usual care control group (n = 28)	Targeted – self selected CBT -psychoeducation -goal setting -time management -cognitive restructuring -problem solving -managing perfectionism and procrastination -preparing for exams	Trained school psychologist or teacher 8 sessions	1. Depression Anxiety and Stress Scale-21 (DASS-21; Lovibond & Lovibond, 1995) 2. Self-Efficacy Questionnaire for Children (SEQ-C) 3. Strengths and Difficulties Questionnaire-Teacher Version (SDQ-TV)	Significant effect for stress at post-treatment (p = 0.002 (g = 0.20)), F(1,38.011) = 10.607	Stress significantly reduced pre- to post-intervention compared to control, and this was maintained at 3-month follow-up. No significant change in anxiety or depression pre- to post-intervention compared to control Self-efficacy (emotional and academic) significantly increased pre- to post-intervention compared to control. Increase in emotional self-efficacy was maintained at 3-month follow-up No significant effects for teacher reported symptoms
8. Puolakanaho et al. [50]	Finnish schools (number of schools not reported)	249	Age not reported Grade 9	1. Online ACT program (n = 82) 2. Online plus face-to-face ACT program (n = 83) 3. No intervention control (n = 84)	Targeted – academically struggling & non-struggling students CBT -setting goals -cognitive and behavioural strategies based around acceptance and diffusion from one’s thoughts and feelings -promoting good relationships with others through practicing compassion	Professional instructors 5 sessions (plus 2 face to face sessions for the online plus face to face ACT program)	1. Overall stress (researcher designed) 2. School stress (Scale adapted from the Health Behaviour in School Aged Children study) 3. Academic Buoyancy Scale (ABS)	Intervention groups combined showed significant decrease in overall stress (p = 0.037, d = 0.22) & no clear indication of changes in school stress (p = 0.057, d = 0.18)	Based on the whole sample (intent-to-treat analyses), the intervention groups did not differ significantly from the control group, in terms of their change in overall or school stress Based on the participants who completed treatment, the intervention groups combined (i.e., online ACT and face-to-Face ACT) experienced a significant decrease in overall stress and increase in academic buoyancy. Further, these changes were significantly greater compared to the control group. No significant change in school stress was observed
9. Putwain & Pescod [51]	2 English schools	56	Age not reported Grades 10 & 11	1. CBT test-anxiety intervention (STEPs) (n = 25) 2. Waitlist control (n = 31)	Targeted – high test anxiety CBT -identifying test anxiety -goal setting -replacing negative self-talk -relaxation -improving test taking	Psychologists/computer 6 sessions	1. Revised Test Anxiety Scale (RTA; [43]	Intervention group showed moderate worry decline t(24) = 4.63, p < 0.001, d = 0.76	Significantly greater reduction in test anxiety from pre- to post-treatment for students in the intervention, relative to control group
10. Putwain & von der Embse (2021)	8 English schools	146	14–16 years. Grades 10 & 11	1. CBT test-anxiety intervention (STEPs) (n = 80) 2. Waitlist control (n = 81)	Targeted – high test anxiety CBT -identifying test anxiety -goal setting -replacing negative self-talk -relaxation -improving test taking skills	Professional instructors/ computer 6 sessions	1. Revised Test Anxiety Scale (RTA; [43] 2. School Related Wellbeing Scale (SWBS) 2. Revised Children’s Anxiety and Depression Scale, general anxiety, and panic subscales (RCADS)	Participants in intervention showed a larger statistically significant decrease in test anxiety: t(64) = 6.75, *p* < 0.001, d = 0.86, and a small decrease in clinical anxiety: *t* (64) = 3.74, *p* < 0.001, d = 0.43	Significantly greater reductions in test anxiety and clinical anxiety (generalised and panic) for students in the intervention, relative to control group
11. Shen et al. [52]	3 Chinese schools	75	Age not reported Grade 11	1. Expressive writing (n = 38) 2. Control writing (n = 37)	Targeted – high test anxiety Expressive writing -Write about positive emotions felt every day	Teachers 30 sessions	1. Test Anxiety Scale for Children (TASC)	Avg. post-test TAS score was significantly lower (F = 135.80; *p* < 0.001)	Significantly lower test anxiety for students in the intervention at post-treatment, relative to control group
12. Sportel et al. [53]	24 Dutch schools	240	12–15 years Grades 7 & 8	1. Internet-based CBM training (n = 86) 2. School-based CBT (n = 84) 3. No intervention control (n = 70)	Targeted – high test or social anxiety in school setting Cognitive bias modification -computer based tasks to modify interpretation and attention biases, strengthen association between social evaluative situations and positive outcomes and increase implicit self-esteem	Psychologist 20 sessions -Computer tasks to modify interpretation and attention biases	1. Revised Child Anxiety and Depression Scale – Social phobia subscale [38] 2. Spielberger Test Anxiety Inventory (TAI)	Significantly stronger reduction of test anxiety scores Pre – post (Cohen’s* d* = 0.32) and post – follow-up (Cohen’s *d* = 0.58)	Significantly greater reduction in test anxiety symptoms for students in the CBT condition between pre- to post-intervention, and post-intervention to 6-month follow-up, relative to control. No significant differences between CBM and control or CBM and CBT No significant difference in social anxiety symptoms pre- to post-intervention between CBM and control, or CBM and CBT. CBT group had a significantly greater reduction in social anxiety symptoms between post-intervention and 6-month follow-up Between pre-test and 1-year follow-up, significantly larger reduction in test anxiety for students in the CBT intervention relative to control. No other significant effects
13. Stanton [54]	1 Australian school	40	12–15 years Grade not reported	1. Self-hypnosis (n = 20) 2. No intervention control (n = 20)	Targeted – high test anxiety Imagery/ Hypnosis -Relaxation focused on breath -Imagery encouraging positive and disposing of unhelpful characteristics -Imagining a special place, and how they would like to be	Psychologist 2 sessions	1. Test Anxiety Scale for Children (TASC; [55]	Significant reduction in text anxiety. (df = 19; F = 12.63, p < 0.01)	Test anxiety significantly reduced for students in the intervention, maintained at 6-month follow-up. No changes for the control group
14. Sud & Prabha [56]	Indian schools (number not reported)	80	Age not reported. Grade 9	1. Attention Skills Training (n = 20) 2. Attention skills control* (n = 20) 3. Relaxation training (n = 20) 4. Relaxation control* (n = 20) *Control groups met with psychologists for one session	Targeted – high- and low-test anxiety Cognitive Bias Modification -Identification of unhelpful cognitions -Plans for approaching tasks -coping self- statements -counters for irrational rumination -self rewarding statements	Not reported 3 sessions	1. Test Anxiety Inventory—Hindi (TAI; [57] 2. Present Affect Reaction Questionnaire (PARQ)	Significant (p < .01) pre to posttreatment reduction in W-Trait of high-test anxious girls only in Attentional Skills Training condition. No effect size given	The worry component of test anxiety significantly reduced from pre- to post-intervention for students in the attentional skills training condition only. This was maintained at 4-week follow-up. No significant change in state anxiety (PARQ) scores. No significant between group differences
15. Van der Ploeg & Van der Ploeg-Stapert [58]	21 Dutch schools	68	12–20 Grades not reported	1. Behaviour-modification program (n = 43) 2. Waitlist control (n = 25)	Targeted – high test anxiety CBT -psychoeducation -relaxation -study skills & time management -self-monitoring -dealing with concentration difficulties -ABC (Activating event, Belief system and emotional Consequences) analysis	Psychologists 8 sessions	1. Spielberger Test Anxiety Inventory – Dutch (TAI) 2. State-Trait Anxiety Inventory – Dutch	significant main effect for treatment versus control group, pre- versus post-test (F = 5.63, p < .001). No effect size given	Significant reduction in test anxiety for the intervention compared to control group pre- to post-intervention. Whereas the control group showed a slight increase in anxiety
16. Weems et al. [59]	5 American schools	1,048	8 – 17 years. Grade 3—12	1. Test Anxiety Intervention (n = 203) 2. Waitlist control (n = 122) 3. No treatment control (n = 723)	Targeted – high test anxiety CBT -psychoeducation (i.e., cognitive behavioural model of anxiety) -exposure related to test anxiety -building self-efficacy related to tests -test taking skills -relaxation strategies	Counsellors 5 sessions	1. Test Anxiety Scale for Children (TASC; [60] 2. Revised Child anxiety and Depression Scale (RCADS; [38] 3. Anxiety Control Questionnaire – Child Version (ACQ-C)	Test anxiety and RCADS anxiety: treatment group sig. higher decrease pre to post, compared to waitlist control (test anxiety: co-efficient = .08, t(307) = 2.82, p < .01 d = 0.84; RCADS: co-efficient = .14, t(303) = 2.27; p < .05, d = 0.34). No sig. interaction for age or grade	Test anxiety, and overall anxiety and depression decreased for the intervention over and above the waitlist control group. These results were not moderated by age or grade level. Further, there was a significant change in perceived anxiety control pre- to post-intervention, however this was only seen for youth in grades 9 and above i.e., older youth Follow-up analyses: Reduction in test anxiety was maintained at two-year follow-up

**Table 2:** Universal interventions included in the review

Author	Setting	Overall sample (N)	Age range/ Grade	Intervention and Comparison groups (n)	Format & treatment type category	Facilitator(s) & number of treatment sessions	Stress/Anxiety Outcome Measure(s)	Results	Findings Summary
1. Frank et al. [61]	2 American schools	251	Mean age 16 years. Grade 11	1. Learning to BREATHE (n = 131) 2. Usual health education class curriculum (n = 124)	Universal Mindfulness/ Meditation -psychoeducation about mindfulness -somatic awareness -automatic self-talk and mindfulness -understanding how emotions effects thoughts and body -stress and stress reactions -cultivating loving kindness	Teachers 12 sessions	1. Generalized Anxiety Disorder Scale (GAD-7; [62] 2. Adolescent Stress Questionnaire (ASQ; [47] including Stress of School Performance & Stress of Peer Pressure subscales	Practice effects for stress of school performance were just short of significant (d = -0.27)	No significant difference between the intervention group and control group pre- to post-intervention for anxiety or stress
2. Gregor [63]	1 English school	105	16 – 17 years. Grade 11	1. Mixed CBT and relaxation (n = 26) 2. Relaxation only (n = 26) 3. CBT only (n = 26) 4. Usual class (PSHE; Personal Social and Health Education) control (n = 26*) *One group (not specified) had 27 participants	Universal CBT -exploring/ challenging faulty thoughts and beliefs -addressing responses to anxiety -positive self-talk -mind mapping -problem solving & coping strategies -taking control of learning and performance	Teachers 15 sessions	1. The Friedben Test Anxiety Scale (FTA; [64] 2. Conners’ Rating Scales – Revised Teacher form (1997) – Anxiety/shy factor	Significant dif in teacher rated anxiety for groups (F(3) = 9.403, p < 0.0005) No significant dif. between groups pre-post treatment	No significant difference in test anxiety between groups pre- to post-intervention, although the CBT alone, relaxation alone and control groups did experience a reduction in test anxiety. Mixed group reported lower level of test anxiety at pre-intervention compared to the remaining three groups Significant difference between groups on teacher rated anxiety: anxiety increased pre- to post-intervention among students in the mixed CBT and relaxation group, whereas it decreased among students in the other three groups
3. Hiebert et al. [65] study 2	2 Canadian schools	113	13—14 years Grade 8	1. Progressive relaxation (n = 62) 2. Control -career education class (n = 51)	Universal Relaxation -Monitoring of heart rate and skin temperature -Progressive muscle relaxation script practice -Cue controlled relaxation -Visualisation	Psychologists 8 sessions	1. State-Trait Anxiety Inventory 2. Psychophysiological Stress Profile (PSP) 3. Symptoms of Stress Inventory (SOSI; [82])	Significantly lower trait anxiety in intervention condition (F(1,41) = 5.57,p = 0.02)	Significantly lower trait anxiety for students in the intervention condition, relative to control. Reduction in stress pre- to post-intervention for both groups
4. Keogh et al. [34]	1 English school	209	15—16 years Grades not reported	1. Cognitive-behavioural stress management intervention (n = 80) 2. No intervention control (n = 80)	Universal CBT -psychoeducation -challenging unhelpful cognitions -progressive muscle relaxation -modify meta-cognitions about worry -adaptive problem solving -guided imagery	Psychologist 10 sessions	1. Revised Test Anxiety Scale (RTA; [43] 2. General Health Questionnaire (GHQ) – Mental ill-Health 3. GCSE points	No significant main, or interactive, effects	No significant changes in test anxiety Mental ill-health improved from pre- to post-intervention for the CBT group but not the control group The CBT group obtained higher GCSE scores compared to the control group, with the CBT group obtaining one letter grade (i.e., B grade) higher than the control group (i.e., C grade)
5. Khalsa et al. [44]	1 American school	121	15—19 years. Grades 11 & 12	1. Yoga (n = 74) 2. Usual Physical Education class (n = 47)	Universal Mindfulness/ Meditation -training in cognitive skills of mindfulness and self-awareness -yoga based psychological attitude -yoga poses -breathing exercises -visualisation -relaxation -stress management	Professional instructor 11 weeks, 2–3 sessions per week (i.e., 23–32 sessions dependent on class)	1. Perceived Stress Scale (PSS) 2. Profile of Mood States-Short Form (POMS-SF) 3. Behaviour Assessment Survey for Children-Version 2 – includes test anxiety scale	No significant group differences (p = 0.15, d = 0.33)	No significant group differences for anxiety or stress pre- to post- intervention
6. Kiselica et al. [45]	1 American school	48	Age not reported Grade 9	1. Stress inoculation training (n = 24) 2. Active control (n = 24)	Universal CBT -psychoeducation -progressive muscle relaxation -cognitive restructuring -assertiveness training	Psychologists 8 sessions	1. State-Trait Anxiety Inventory – Trait (STAI A-TRAIT; [85]) 2. Symptoms of Stress Inventory (SOSI; [82]) 3. GPA	Significant dif. between treatment and control participants (F(1,41) = 14.04, p < 0.001, η^2^ = 0.25)	Significantly lower anxiety and stress for students in the intervention condition compared to the control condition at post-treatment and follow-up. No significant differences between groups for GPA
7. Lang et al. [46]	1 Swiss vocational school	131	Mean age 16.22 years Age range and grade not reported	1. PE based coping training -EPHECT (n = 67) 2. No intervention control (n = 64)	Universal CBT -psychoeducation -emotion and problem focussed coping skills	Teachers 8 sessions	1. Adolescents Stress Questionnaire (ASQ; [47]	Time by group interactive effect at follow- up (F(1,111) = 3.82, p < 0.05, η^2^ = 0.035)	Significantly greater reduction in stress for the intervention group, relative to controls, at 6-month follow-up
8. Lang et al. [48] *Same sample as 2016	1 Swiss vocational school	131	Mean age 16.22 years Age range and grade not reported)	1. PE based coping training -EPHECT (n = 67) 2. No intervention control (n = 64)	Universal CBT -psychoeducation -emotion and problem focussed coping skills	Teachers 8 sessions	1. Adolescents Stress Questionnaire (ASQ; [47] 2. Coping Questionnaire for Children and Adolescents (SVF-KJ)	No significant changes pre-post treatment	Intervention group reported greater increase in emotion focused coping compared to control group pre-post intervention No significant changes in perceived stress from pre- to post-treatment
9. Putwain et al. [66]	10 English schools	3225	14—16 years Grades 9, 10 & 11	1. CBT test-anxiety intervention (STEPs) (n = 1600, only 624 completed all or some of the program) 2. Waitlist control (n = 1625)	Universal CBT -identifying test anxiety -goal setting -replacing negative self-talk -relaxation -improving test taking	Teachers/ computer 6 sessions	1. Revised Test Anxiety Scale (RTA; [43] 2. Friedben Test Anxiety Scale – Social Derogation (FTAS; [64]	Statistically significant declines in worry: t(329) = 4.89, p < 0.001, d = 0.63 and tension: t(329) = 3.46, p = 0.001	Significantly reduced test anxiety for highly test anxious students in the intervention group, from pre- to post-intervention. No significant changes in test anxiety for students with low or mild test anxiety, or for students who did not complete the intervention
10. Stanton [67]	2 Australian schools	60	13 years Grade not reported	1. Guided imagery program (n = 30) 2. Waitlist control (n = 30)	Universal Imagery/ Hypnosis -Relaxation focused on breath -Imagery encouraging positive and disposing of unhelpful characteristics -Imagining a special place, and how they would like to be	Psychologist 3 sessions	1. Student Stress Inventory	Significant stress reduction (*t* = 6.32: *df* = 29; p < 0.001)	Significant reduction in stress reported by students at post-intervention and at 6-month follow-up. No significant reduction for the control group
11. Szabo & Marian [35]	2 Romanian schools	191	15–17 Grades not reported	1. Stress Inoculation Training (n = 64) 2. Counselling (n = 65) 3. No treatment control (n = 62)	Universal CBT -psychoeducation -cognitive appraisal -problem solving -personal experiments including imagery, role play, behavioural reversal and modelling	Psychologists 10 sessions	1. Perceived Stress Questionnaire (PSQ) 2. State-Trait Anxiety Inventory (STAI; [84])	Participants receiving SIT training reported lowest levels; (PSQ: F (2,188) = 361.08; p = .001; STAI: F(2,188) = 553.26; p = .001)	Participants in the intervention group reported significantly lower anxiety and perceived stress post-intervention compared to the counselling and control groups At three-month follow-up, participants in the intervention group reported significantly lower perceived stress compared to the counselling and control groups
12. Venturo-Conerly et al. [68]	2 Kenyan schools	895	14 – 18 years. Grades not reported	1. Growth intervention (n = 240) 2. Gratitude intervention (n = 221) 2. Value Affirmation intervention (n = 244) 3. Control intervention – Study Skills (n = 190)	Universal Cognitive bias modification Growth: -discussed neuroplasticity and growth in face of challenges -growth strategy implementation e.g., problem solving -helping someone else grow Gratitude: -discussion of feeling gratitude -gratitude letter- thanking someone -writing three things they felt gratitude for each day for one week Value Affirmation: -Selected and wrote about a chosen value -completed a value promoting activity at home	Lay providers i.e., recent high school graduates 1 session with an at home activity	1. General Anxiety Disorder screener – 7 (GAD-7; [62] 2. Patient Health Questionnaire – 8 (PHQ-8)	Clinical sample: significant time*condition interaction effect on anxiety symptoms favouring the values condition (B = − 2.22, p < .01; Cohen’s d = 0.49 [0.09–0.89]) and the growth condition (B = − 1.78, p < .05; Cohen’s d = 0.39 [0.01–0.76])) over the control	Whole sample: Significant reduction in anxiety pre- to post-intervention among students in the Value Affirmation group compared to the control group Clinical subsample: Significant reduction in anxiety pre- to post-intervention among students in the Value Affirmation and Growth groups compared to the Control group
13. Wang et al. [69]	70 Chinese schools	7,495	Age not reported. Grades 7 & 8	1. Social Emotional Learning Program (n = 3,694) 2. No intervention control (n = 3,801)	Universal Social Emotional Learning - emotion management -self-awareness -setting goals -establishing positive relationships	Teachers 32 sessions	1. Learning Anxiety Index, 15 questions from the Mental Health Test (MHT)	Students who received the SEL program were 0.061 SDs more likely to choose more appropriate responses to such challenges (p = 0.008)	Significant reduction in school dropout and learning anxiety symptoms among the SEL compared to control group at 8-months post baseline assessment (post-intervention). However, this was not maintained at follow-up (15-months post baseline) For at risk students (i.e., older students and those who kept in touch with students who had dropped out of school) there was a significant reduction in school dropout and learning anxiety from pre- to post-intervention and this was maintained at follow-up
14. Weems et al. [70]	1 American school	94	13 – 16 years. Grade 9	1. Test Anxiety Intervention (n = 16) 2. Waitlist control (n = 14) 3. No treatment control (n = 64)	Universal CBT -psychoeducation (i.e., cognitive behavioural model of anxiety) -exposure related to test anxiety -building self-efficacy related to tests -test taking skills -relaxation strategies	Psychologist 5 sessions	1. Test Anxiety Scale for Children (TASC; [60] 2. Grade Point Average (GPA) results	Treatment group: [t(15) = 5.05, p b .001, p rep = .998, 90% C.I. for the mean difference 2.4 to 4.9, d = 1.2]	Significantly reduced state and test anxiety for students in the intervention, relative to control groups GPA significantly increased for both intervention and waitlist groups, but the effect size was higher for the intervention group
15. Yahav & Cohen [71]	2 Israeli schools (1 Jewish, 1 Arab)	255	14–16 years Grade 9	1. Cognitive-behavioural stress management training (n = 126) 2. No intervention control (n = 129)	Universal CBT -identification of stress and common reactions -psychoeducation -cognitive model of stress and thought restructuring -relaxation with biofeedback, progressive muscle relaxation and imagery	Psychologists 8 sessions	1. State-Trait Anxiety Inventory – State subscale [87] 2. Spielberger Test Anxiety Inventory -(TAI; [57], adapted by Zeidner)	Test anxiety: Intervention group sig. decrease compared to control group (F(1, 223) = 6.39, p < .01). State anxiety: Intervention group sig. decrease compared to control group for Arab school only (Group x school interaction: F(1, 223) = 5.93, p < .05)	Significant decrease in test anxiety for students in the intervention group, compared to students in the waitlist group pre- to post-intervention. This effect was more prominent for Arab compared to Jewish students. For state anxiety, significant decrease pre- to post-intervention seen only for the Arab students

### Methodological Quality

The quality of studies was evaluated by two authors using the Critical Appraisal Skills Programme (CASP, 2018), which includes a checklist for rating cohort studies. Based on this checklist, studies were scored according to five quality criteria; (a) explored a focused issue; (b) included an appropriate sample; (c) used outcome measures that were unlikely to be biased; (d) used an appropriate design and considered confounds; (e) analysed and interpreted results appropriately. Table 3 presents the quality ratings for each study, as meeting or not meeting the criteria (or unclear).

**Table 3:** Quality ratings of articles included in the review

Author	Focused issue	Adequate sample	Unlikely measurement bias	Appropriate design/ confounds considered	Adequate analysis/ interpretation
*Targeted*					
1. de Hullu et al. [37]	Yes	Yes	Yes	Yes	Yes
2. Gaesser & Karan [39]	Unclear	No	Yes	No	Unclear
3. Hains & Ellman [40]	Yes	No	Yes	Yes	Yes
4. Johnson et al. [41]	Yes	No	No	Yes	Yes
5. Kamour & Altakhayneh [42]	Yes	Yes	No	No	No
6. Laxer & Walker [49]	Unclear	Yes	Yes	Unclear	Unclear
7. Lowe & Wuthrich [83]	Yes	Yes	Yes	Yes	Yes
8. Puolakanaho et al. [50]	Yes	Yes	Unclear	Yes	Yes
9. Putwain & Pescod [51]	Yes	Yes	Yes	Yes	Yes
10. Putwain & von der Embse (2021)	Yes	Yes	Yes	Yes	Yes
11. Shen et al. [52]	Yes	Yes	Yes	Yes	Unclear
12. Sportel et al. [53]	Unclear	Yes	Yes	Yes	Yes
13. Stanton [54]	No	No	Yes	No	Yes
14. Sud & Prabha [56]	No	No	Yes	No	Yes
15. Van der Ploeg & Van der Ploeg-Stapert [58]	Yes	No	Unclear	No	No
16. Weems et al. [59]	Yes	Yes	Yes	Yes	Yes
*Universal*					
1. Frank et al. [61]	Yes	Yes	Yes	Yes	Yes
2. Gregor [63]	Yes	No	Yes	Yes	Yes
3. Hiebert et al. [65]	No	Unclear	Unclear	Unclear	Yes
4. Keogh et al. [34]	Yes	Unclear	Yes	Yes	Yes
5. Khalsa et al. [44]	No	Unclear	Yes	No	No
6. Kiselica et al. [45]	Yes	Unclear	Yes	Unclear	Unclear
7. Lang et al. [46]	Yes	Unclear	Yes	Yes	Yes
8. Lang et al. [48]	Yes	Unclear	Yes	Yes	Yes
9. Putwain et al. [66]	Yes	Yes	Yes	Yes	Yes
10. Stanton [67]	No	Unclear	No	Unclear	Yes
11. Szabo & Marian [35]	Yes	Yes	Yes	Yes	Yes
12. Venturo-Conerly et al. [68]	Yes	Yes	Yes	Yes	Yes
13. Wang et al. [69]	No	Unclear	Yes	Unclear	Unclear
14. Weems et al. [70]	Yes	No	Yes	Yes	Yes
15. Yahav and Cohen [71]	Yes	Yes	Yes	Yes	Yes

Yes = met criterion, No = did not meet criterion, Unclear = unclear if criterion was met

## Results

Studies were conducted across 13 different countries: United States of America (n = 8), United Kingdom (n = 4), Australia (n = 3), Canada (n = 3), Netherlands (n = 3), China (n = 2), Switzerland (n = 2), Finland (n = 1), India (n = 1), Israel (n = 1), Jordan (n = 1), Kenya (n = 1) and Romania (n = 1). Seventeen studies evaluated a targeted intervention whereas 14 studies evaluated a universal intervention.

### Outcome Measures

A wide variety of self-report measures were used to assess changes in academic stress and anxiety across studies. The most used measure included the State-Trait Anxiety Inventory (STAI; [87]) which was used in six studies to measure anxiety [35, 40, 45, 58, 65, 71]. This was followed by the Spielberger Test Anxiety Inventory (TAI [57], or a variation of this measure, used in five studies [37, 53, 56, 58, 71]. The Revised Test Anxiety Scale (RTA [43], and Test Anxiety Scale for Children [60], 1978, [55] were used in four studies. Measures used less frequently were the Adolescent Stress Questionnaire (ASQ [47], the Friedben Test Anxiety Scale (FTAS [64], Revised Childrens Anxiety and Depression Scale (RCADS [38], Symptoms of Stress Inventory [82] and the 7-item Anxiety Scale (GAD-7 [62], which were used in two or three studies each. There were several other measures that were used in one study, as seen in Tables 1 and 2.

### Intervention Format and Type

The 31 studies included in the review were categorised as *targeted* and *universal* by JB and TJ. Targeted interventions were run with a select cohort of students who typically scored above a certain cut-off indicating they were experiencing elevated symptoms or ‘vulnerability’ to anxiety or stress. Whereas universal interventions were run with all students regardless of their symptomology. Further, the primary treatment being evaluated in each study was classified into *treatment type*, this decision was made for each study according to the description of the intervention outlined by authors. The treatment type category, as well as a summary of treatment components, for each study can be seen in Tables 1 and 2. Overall, the majority of the 31 studies evaluated programs that included teaching students cognitive and behavioural skills (i.e., CBT, 17 studies) including traditional CBT, as well as third wave CBT. Of the remaining studies, four examined Cognitive Bias Modification, and two studies each examined Mindfulness/Meditation (including hypnosis), Systematic Desensitisation, and Social Emotional Learning. Expressive Writing and Relaxation were evaluated by one study each. The outcomes of each study will be discussed below.

### Targeted Interventions

There were 16 studies identified that targeted a particular sample of participants. Nine of these studies targeted a sample of students with high-test anxiety, three targeted a sample of students with high social anxiety and one study targeted students with a combination of high test and social anxiety (see Table 1). The remaining studies targeted a self-selected sample ([40, 83], or sample with academic strengths or difficulties [39, 50], and one study did not list why they deliberately chose the sample [42].

#### Cognitive Behavioural Therapy

CBT focuses on modifying unhelpful thoughts, beliefs and behaviours known to maintain stress or anxiety. Studies were categorised as CBT if the primary intervention targeted both behaviours and cognitions to reduce academic stress or anxiety. Eight of the targeted programs used CBT including one Acceptance and Commitment Therapy (ACT; [50], one Stress Inoculation Training (SIT [40], and one Behaviour Modification program that also included a focus on cognitions [58]. Of these eight studies, six found a significant reduction in students’ academic stress or anxiety immediately following CBT, compared to a waitlist control group with studies reporting large effect size reductions on measures of test anxiety [51, 58, 59, 72] and small effect size reductions on measures of general stress [83, 50]. Further, follow-up treatment effects were found to be sustained two months post-treatment [40] and three months post treatment [83, 58], however Van der Ploeg and Van der Ploeg-Stapert [58] did not report any statistical analyses for their finding, and no effect sizes were reported for any of the follow-up comparisons. Studies have also indicated CBT programs specifically targeted at reducing academic stress or anxiety have led to a decrease in symptoms of clinical disorders such as panic disorder and generalised anxiety disorder [72] as well as posttraumatic stress disorder [59]. The above findings have relied on self-report measures, [83] included a third-party measure of student symptoms i.e., a teacher measure, however they failed to detect a change in teacher reported emotional problems among students post treatment. The study that did not find a significant change in academic stress or anxiety for the CBT group post-treatment when compared to the waitlist control group [39] also trialled emotional freedom technique (EFT,tapping acupuncture points, and in contrast found the EFT group had a significantly greater change in anxiety pre- to post-intervention compared to waitlist control. Gaesser and Karan [39] acknowledged that their study was underpowered and that the lack of significant benefits for CBT might have been due to insufficient treatment dosage with only three treatment sessions delivered.

#### Systematic Desensitisation

Systematic desensitisation, which involves engaging in relaxation methods whilst visualising stressful scenarios, was analysed as a targeted approach in two studies [41, 49]. Johnson et al. [41] focused on speech anxiety within the school context, whereas Laxer and Walker [49] focused on test anxiety, however both studies yielded positive results. Johnson et al. [41] found that the systematic desensitisation group and speech practice groups both experienced a significant decrease in speech anxiety post-treatment, compared to the no-treatment control group. Laxer and Walker [49] analysed four treatment groups (i.e., systematic desensitisation, relaxation alone, simulation alone, relaxation plus simulation), and compared these four treatment groups to an active control and non-active waitlist control. They found that the students in the systematic desensitisation and relaxation alone conditions experienced a significantly greater decrease in test anxiety compared to the students in the no treatment control condition at post-treatment.

#### Cognitive Bias Modification

The effectiveness of a cognitive-bias modification (CBM) program was evaluated in two randomised controlled trials [37, 53]. Sud and Prabha [56] split their sample into those with high versus low test anxiety and compared the effectiveness of a three-session attention skills training (focused on modifying worry related to test anxiety) to relaxation training as well as two matched control groups. They found that high test anxiety participants in the attention skills training condition experienced a significant decrease in worry associated with test anxiety, and this was maintained at 4-week follow-up. However, there were no significant between group differences and no significant effects on state anxiety. Results from the second CBM trial were published in two papers [37, 53]. de Hullu et al. and Sportel et al.’s CBM program consisted of a 10-week (2 × per week) computer-based intervention with tasks to shift students’ attention and interpretation biases. Results from Sportel et al. [53] revealed that test anxiety or social anxiety levels of students who received CBM were not significantly lower than students in a comparison CBT program (10 group sessions), or a no-intervention control condition, at any timepoint. In fact, CBT led to significantly greater reductions in test anxiety from pre-treatment to post-treatment, as well as 6-month and 12-month follow-up compared to no-treatment control. These results suggest that CBT was more beneficial than no-treatment, whereas CBM was not. However, de Hullu et al. [37] reported that the difference in symptoms for the CBT group compared to the CBM and control group, on measures of test anxiety and social anxiety, were no longer significant at 2-year follow-up. These studies suggests that treatments focused on modifying unhelpful cognitions alone, do not appear to be more effective in treating academic related stress or anxiety.

#### Other Programs

Other targeted programs that reported effective in treating academic stress or anxiety, were only examined in a single study. One randomised study provided some evidence that self-hypnosis significantly reduced test anxiety among highly test anxious students, compared to no intervention, and that this effect was maintained at 6-month follow-up [54]. Further, Shen et al. [52] reported that writing about positive emotions everyday led to a significantly greater decrease in test anxiety compared to neutral writing. One further study by Kamour and Altakhayneh [42] found that treatment aimed at improving social emotional learning, i.e., developing emotional intelligence related to school, led to a decrease in maths anxiety, however the quality rating for this study was poor (see Table 2).

### Control Conditions

Most (i.e., 13 out of 16) targeted studies included a *no intervention* or *waitlist* control group [37, 39–41, 49, 83, 50, 51, 53, 54, 58, 59, 72]. Of these 13 studies, 11 found significant between group differences in favour of the treatment group, or a significant change in symptoms for the treatment group, but no effect for the control group. Of the two studies that found no effect between groups, one implemented CBM [37] and the other CBT [40]. Six studies [37, 39, 41, 49, 50, 53] included alternative treatment groups as well as an inactive control group, and three of these compared online and face to face programs [37, 50, 53]. Of these six studies, none found a significant difference between target treatment and active control groups, although Laxer and Walker [49] did not report differences between active treatment groups as they focused on differences between treatment and inactive control groups. Three studies only included an active control group with no inactive control [42, 52, 56], and all three found significant differences in favour of the treatment compared to control group. Although, Sud and Prabha [56] did not find significant between group differences.

### Universal Interventions

There were 15 studies that evaluated treatments to reduce academic stress and anxiety among a non-selected (universal) sample of students. See Table 2.

#### Cognitive Behavioural Therapy

Nine of the studies evaluating universal interventions examined the effectiveness of CBT [34, 35, 45, 46, 63, 66, 70, 71]. Compared to results of studies investigating targeted CBT, studies investigating universal CBT were more mixed, with seven of the nine studies reporting a reduction in academic stress and anxiety post intervention or at follow-up [35, 45, 46, 48, 66, 70, 71], however one of these studies only reported a reduction in symptoms for students with high test anxiety [66].

Researchers have found that students who complete CBT as part of a universal treatment report lower anxiety and stress compared to control participants at post-treatment, as well as 4-week [45] and 3-month follow-up [35]. Effect size benefits were generally reported to be moderate. Further, although Lang et al. (2016, 2017 did not find a reduction in stress among students immediately following CBT, they reported a significant increase in emotion focused coping skills, and a reduction in stress relative to the control group at 6-month follow-up. Further, the effects of CBT aimed at reducing academic stress and anxiety may generalise to other psychological symptoms. Weems et al. [70] also examined test anxiety pre- to post-CBT among children exposed to Hurricane Katrina, they found a reduction in test anxiety in the treatment group compared to wait-list control. Further, Weems et al. [70] reported that PTSD symptoms within the CBT group significantly decreased, whereas those in the waitlist control group did not. However, Yahav and Cohen [71] examined the effect of CBT on test anxiety and state anxiety among Israeli Jewish and Israeli Arab students and they found that students in the CBT condition experienced a decrease in these symptoms compared to controls pre- to post-treatment. Although, the effect on test anxiety was most pronounced among Arab compared to Jewish students, possibly due to their higher reported test anxiety pre-treatment.

Other studies have been more mixed in terms of their findings regarding the effectiveness of universal CBT programs. Putwain et al. [66] found students with high test anxiety reported a reduction in test anxiety post CBT, however those with mild to low test anxiety did not. Further, Gregor et al. (2005) conducted a study evaluating the effectiveness of CBT alone, relaxation alone and a mix of CBT and relaxation, compared to a control condition. They found no significant difference between student reported anxiety pre- to post-treatment among students in these four conditions. However, they also included a teacher measure of anxiety and found that teachers rated students in the relaxation alone group as significantly less anxious compared to the CBT alone or control group. On both student and teacher measures, students in the mixed CBT and relaxation group had an *increase* in anxiety post-treatment, however these students started with lower anxiety ratings compared to the other groups indicating that CBT may be more beneficial for those with higher test anxiety, like findings from Yahav and Cohen [71] and Putwain et al. [66]. In line with findings from Gregor et al. (2005), Keogh et al. [34] also did not find that their CBT program decreased test anxiety, however they did report a decrease in mental ill-health among their sample following treatment compared to the control group.

#### Mindfulness and Meditation Programs

Mindfulness as a universal program was analysed in two studies [44, 61]. Results from these studies indicate that mindfulness/meditation interventions aimed at reducing academic stress and anxiety were no more effective at reducing stress and anxiety symptoms compared to an active control condition. Frank et al. [61] reported no significant difference between the mindfulness group and usual health education control group for symptoms of anxiety or stress pre- to post-intervention. Further, Khalsa et al. [44] compared a yoga intervention with a focus on mindfulness to usual physical education class and reported no significant difference in anxiety or stress between these groups pre- to post-treatment.

#### Other Programs

Four authors analysed another type of intervention among a universal sample. Although these interventions are encouraging, they were only evaluated in one study each. Stanton [67] conducted a study of imagery/hypnosis compared to a no-treatment control, he reported that the intervention group experienced a decrease in self-reported stress pre- to post treatment, which was maintained at 6-month follow-up, whereas there was no change in stress for the control group over time. Hiebert et al. [65] randomly allocated students across Grade 8 to progressive muscle relaxation or an active control group (career education class), they reported that progressive muscle relaxation led to a reduction in trait anxiety compared to the active control condition. Wang et al. [69] implemented a social emotional learning intervention over 32 sessions among a large number of Chinese students (3,694 students) and compared this to a no-intervention control condition. They found a significant reduction in learning anxiety symptoms and school dropout among students in the intervention condition compared to control at post-treatment, but this reduction was not maintained at the 6-month follow-up. Another more recent study by Venturo-Conerly et al. [68] examined a novel one-treatment session approach that targeted cognitions across three conditions i.e., growth, gratitude, value affirmation, and compared these to a control condition i.e., study skills. They found that students in the value affirmation condition reported lower anxiety compared to the control intervention two weeks post intervention, however the other interventions had no effect on anxiety relative to the control condition.

#### Control Conditions

Of the 15 universal studies, 12 included *no intervention* or *waitlist* control groups, whilst three studies included active control groups only [45, 65, 68]. When compared to no intervention or waitlist control groups, most studies (i.e., 9) found a significant difference between groups, or a significant reduction in symptoms among treatment groups but no reduction for control participants. The three studies that found no such differences trialled CBT [34] and Mindfulness/meditation [44, 61]. Of the 12 studies that included no intervention or waitlist control groups, two also included active control groups [35, 63], however only Szabo and Marian [35] found a significant effect between the treatment and both active and inactive control groups, in favour of the target treatment group. Of the three studies that included active control conditions only, two trialled CBT (i.e., [45, 68] and one trialled PMR (i.e., [65], and all three studies found significant between group differences.

### Intervention Facilitator

In 15 of the 31 studies, programs were delivered solely by psychologists or counsellors (school staff or external), 11 of the 15 studies were targeted rather than universal samples, and 10 of the 15 studies included CBT interventions as their primary treatment. All but one of the studies that were delivered by psychologists or counsellors reported significant reductions in anxiety or stress pre- to post-intervention. However, the study by Keogh et al. [34] that did not report a reduction in test anxiety reported a reduction in mental ill-health for students in the intervention group. Teachers delivered the program exclusively in six out of the 31 studies, five of these six studies were universal rather than targeted samples, and their modality was mixed, with three of the six studies including CBT as the intervention, and the other three studies including expressive writing, social and emotional learning, and mindfulness/meditation. Two of the six studies reported change in academic anxiety or stress pre- to post-intervention and these studies included expressive writing [52] social and emotional learning [69] interventions. One study reported a significant reduction in stress for the intervention compared to control group at 6-month follow-up [46, 48], and one study reported significant reduction in test anxiety for highly test anxious students in the intervention group compared to control group [66], and both studies included a CBT based intervention. One study reported psychologists *or* teachers delivering CBT with a reduction in stress, and an increase in self-efficacy from pre- to post intervention for the intervention group. Professional instructors delivered a program in five studies and these programs were either CBT [39, 50, 72], mindfulness/meditation [44] or systematic desensitisation [49]. Such programs were effectively delivered to result in significant reduction in academic anxiety or stress in all but one study (i.e., mindfulness/meditation,[44]. In one study [68] lay providers delivered the program with limited success. Two studies [42, 56] did not report the facilitator of the program.

### Methodological Quality

The methodological quality of studies was varied, and only 10 of the 31 studies were of high quality i.e., meeting all five criteria (see Table 3). The most common problem was that studies lacked an adequate sample, for example they may have only sampled one class or school, which limits generalisability of their results (e.g., [40, 46, 48]. Some authors did not outline their aims, hypotheses, or primary outcome measures (e.g., [39, 69], some used unvalidated measures (e.g., [50], and some did not consider potential confounds such as gender, school, timing of assessments (e.g., [44, 65]. A few studies did not report enough detail in their results, for example the significance level of changes in means (e.g., [42, 58], or adjust the p-value for the number of tests performed (e.g., [44]).

## Discussion

This systematic literature review aimed to examine the effectiveness of school-based academic stress programs in high school students. The review also aimed to understand delivery characteristics that may alter program effectiveness. In general, the results suggest that CBT programs delivered as a targeted approach had the most benefit, with large effect sizes reductions in test anxiety and small effect size reductions in general stress. The effectiveness of universal programs was more mixed, with the most evidence for CBT interventions which were associated with moderate effect size benefits. Although there was some preliminary evidence for programs using other interventional methods (e.g., systematic desensitisation, expressive writing), more research is needed to establish their efficacy.

In general, there was more support for interventions that used psychologists to deliver the program. However, this result may be conflated with the theoretical underpinnings of these interventions, which were typically CBT-based. There was some evidence that teachers were able to deliver programs successfully, and while it is possible that some teacher-led programs were ineffective due to low implementation fidelity (see [26], more research is needed to examine how adequately teachers adhered to the programs.

### Universal Versus Targeted Approaches

Overall, results showed that both universal and targeted approaches to delivering academic stress programs can be beneficial. This is consistent with meta-analyses finding comparable effect sizes for universal and targeted school-based programs for anxiety disorders [29]. However, careful analysis of the included studies suggests that targeted treatment may be slightly more efficacious (e.g., [71]. Considering the pros and cons of each approach along with their unique implementation issues may assist schools in choosing whether to run a universal or targeted program with their students (see [26, 29].

Universal programs have the appealing potential to help students who are already highly distressed and, at the same time, prevent distress from increasing to clinical levels in the future for other students. Not needing to screen students means less resources are required, and negative stigma is reduced (because students are not singled out). An inherent difficulty with universal programs, however, is that not all students will be distressed and require intervention. Which may be why results from randomised studies are not as strong i.e., these results are watered down as the intervention does nothing for these students because their distress is already low. Further, disengagement and drop-out may be likely for students who perceive the program to be irrelevant to them. A challenge for facilitators, then, is how to engage these students. A notable challenge is that if the program is to be facilitated by teachers, then a whole-school approach will require all school staff to be on board with the program, trained, supervised, and consistent in their delivery.

Targeted programs are usually delivered to a smaller number of students and therefore require fewer trained facilitators. As such, targeted programs can feasibly be delivered by external providers to place less demand on teachers. If time is an issue, targeted programs can be run as an after-school class, which would also be less disruptive. Some schools may choose targeted programs if they address specific difficulties that are prevalent in their student population, as opposed to universal programs that may address more general difficulties. Conversely, the targeted approach has some drawbacks. School staff may not have the expertise to identify symptoms of stress or anxiety in their students, and therefore may require more training. Moreover, teachers who facilitate targeted programs may not be adequately equipped to manage high-risk students and will likely need additional support.

### Study Limitations

Conclusions drawn from the current review should be considered in light of its limitations. First, our search was limited to articles published in English, which meant most studies were conducted in developed countries. Therefore, the suitability of programs and schools’ access to resources necessary for their delivery may be different in developing countries, other international education systems or other cultures. Second, by limiting our results to studies published in peer-reviewed journals, it is possible that other publications of academic stress programs (e.g., in book chapters, school journals or educational reports) were not considered.

The overall quality of the studies included in this review was variable, with several weaknesses which limit the conclusions that can be made. Most studies compared the intervention to an inactive control (no intervention or waitlist) and so it is unclear if these interventions were more beneficial than non-specific treatment effects (Gallin & Ognibene, 2012). Only one of the targeted CBT interventions was compared to an active control group [39] and as such it is not clear whether CBT programs are better than active controls. Further, in most cases targeted intervention studies recruited participants who volunteered that they felt distressed rather than using cut-off scores to enrol only those students who had heightened symptoms. Other factors may also have created variability in the study’s results, such as the outcome measures used and the level of allocation to conditions (i.e. classes versus schools). Overall, this field of research will benefit from more high-quality studies that use random allocation, adequate sample sizes, validated measures, and comparison to active control conditions.

### Future Directions

In line with theoretical models of academic stress, the findings support our hypothesis that the interventions most likely to be effective were programs that targeted known factors underlying and maintaining academic stress, namely irrational thoughts, and unhelpful study behaviours. While the CBT programs addressed some of the underlying factors, increased efficacy might come from more structured targeting of factors specific to academic stress, such as perfectionism and procrastination. Further improvements might come from integrating feedback from students or teachers. This could yield important information, such as whether certain strategies/skills are helpful, which could help to refine programs to their essential components and make them easier for teachers to deliver. Better screening tools with validated cut-offs are also needed to help school staff identify which students would benefit from targeted programs, as the targeted studies in this review were inconsistent in how they determined students with “high” stress/anxiety.

Given sufficient evidence base for CBT programs in targeting academic stress, future research should examine the implementation success in order to improve the rate of successful delivery and uptake as part of routine school activities. Implementation issues (e.g., adherence to a manual, consistency between facilitators, delivery style, and student engagement) can limit a program’s effectiveness [24]. Most of the studies included in the current review did not assess the program’s implementation success. Future researchers may do so using an implementation framework (for an example, see [79]). Some researchers have already done this for school-based CBT programs [81], physical activity programs [77, 78] and mindfulness/Yoga programs [80], however more studies are needed.

It would also be interesting to examine other factors that may influence the effectiveness of academic stress programs, such as the timing of their delivery (i.e., to students in earlier versus later grades). For example, it would be useful for schools to know if programs are more relevant/beneficial when provided to students approaching the high-stakes assessments of their final year, a period typically associated with increased distress [4, 73]. This should also include measurement and reporting of adverse events and drop-outs associated with different interventions. Furthermore, given that few studies in this review conducted a long-term follow-up, future studies should assess effectiveness over time to determine whether booster sessions are needed for students to maintain stress-management skills throughout high school.

## Summary

This systematic literature review focused exclusively on school-based programs designed to reduce or prevent academic stress in high school students who are more likely to experience heightened distress due to increased academic pressures (such as high-stakes assessments). The findings showed that a variety of programs exist but more high-quality studies are needed. The best evidence was for programs grounded in cognitive-behavioural therapy, supporting theoretical understandings of the factors that maintain and exacerbate academic stress. While universal and targeted approaches are both likely to be beneficial, more research is needed to understand how the implementation success of these programs can be improved, particularly when delivered by teachers.

## Data Availability

All data is available in the published literature of the primary source. The composition of the data is available from the authors.
